# Time-expanded phase-sensitive optical time-domain reflectometry

**DOI:** 10.1038/s41377-021-00490-0

**Published:** 2021-03-09

**Authors:** Miguel Soriano-Amat, Hugo F. Martins, Vicente Durán, Luis Costa, Sonia Martin-Lopez, Miguel Gonzalez-Herraez, María R. Fernández-Ruiz

**Affiliations:** 1 Departamento de Electrónica, Universidad de Alcalá, Escuela Politécnica Superior, 28805 Madrid, Spain; 2 Instituto de Óptica “Daza de Valdés”, IO-CSIC, C/Serrano 121, 28006 Madrid, Spain; 3 GROC-UJI, Institute of New Imaging Technologies, University Jaume I, 12071 Castellón, Spain

**Keywords:** Frequency combs, Imaging and sensing

## Abstract

Phase-sensitive optical time-domain reflectometry (ΦOTDR) is a well-established technique that provides spatio-temporal measurements of an environmental variable in real time. This unique capability is being leveraged in an ever-increasing number of applications, from energy transportation or civil security to seismology. To date, a wide number of different approaches have been implemented, providing a plethora of options in terms of performance (resolution, acquisition bandwidth, sensitivity or range). However, to achieve high spatial resolutions, detection bandwidths in the GHz range are typically required, substantially increasing the system cost and complexity. Here, we present a novel ΦOTDR approach that allows a customized time expansion of the received optical traces. Hence, the presented technique reaches cm-scale spatial resolutions over 1 km while requiring a remarkably low detection bandwidth in the MHz regime. This approach relies on the use of dual-comb spectrometry to interrogate the fibre and sample the backscattered light. Random phase-spectral coding is applied to the employed combs to maximize the signal-to-noise ratio of the sensing scheme. A comparison of the proposed method with alternative approaches aimed at similar operation features is provided, along with a thorough analysis of the new trade-offs. Our results demonstrate a radically novel high-resolution ΦOTDR scheme, which could promote new applications in metrology, borehole monitoring or aerospace.

## Introduction

Distributed optical fibre sensing (DOFS) is currently a mature technology capable of monitoring different physical parameters (such as temperature, strain, pressure and birefringence) over long distances, often in the range of tens of kilometres. This technology offers the unique advantage of employing a single sensing fibre to interrogate a large number of points, thus providing a cost-effective solution for the real-time monitoring of large civil structures and long oil and gas pipelines. Conceptually, a DOFS system can be understood as a multiplexed array of sensors working in reflection, where the sensor size becomes an infinitesimal length of fibre and the light is back-reflected due to light scattering (either Rayleigh, Brillouin or Raman). The constant growth and dissemination of DOFS over the last few decades has led to a panoply of techniques and applications, along with a progressive improvement in the sensing performance parameters (namely, the spatial resolution, sensing range, acquisition speed and measurement accuracy)^1–3^.

Among the wide variety of DOFS approaches, phase-sensitive optical time-domain reflectometry, usually abbreviated as ΦOTDR, is a technique based on Rayleigh scattering that stands out for providing the basis of distributed acoustic sensing, that is, the dynamic sensing of vibrations/intrusions along a fibre^4,5^. In ΦOTDR, highly coherent pulses propagating inside a test fibre experience elastic scattering such that the back-reflected signal, after being photo-detected and time resolved, provides a measurement of variations in the temperature or strain along the fibre length. Remarkably, when ΦOTDR is assisted by distributed Raman/Brillouin amplification, sensing ranges beyond 100 km can be reached^6,7^. However, a fundamental limitation in ΦOTDR is the fact that the relationship between the detected amplitude and the strain/temperature variations is, in principle, non-linear. To resolve this drawback, several ΦOTDR approaches have been reported, including ΦOTDR with coherent detection (in which the backscattered signal is mixed with a reference signal)^8,9^, frequency-scanning ΦOTDR^10,11^ and chirped-pulse ΦOTDR^12,13^.

In sensing schemes based on ΦOTDR, the attainable spatial resolution scales inversely with the employed pulse width, being typically 1 m per 10 ns of pulse duration. As a result, high spatial resolutions (in the centimetre range) imply short pulses and, hence, high detection bandwidths, from hundreds of MHz to several GHz. The impact of this requirement on the cost and power consumption of the system can be alleviated by using optical sampling techniques, which combine sensing pulses with an optical gate to dramatically reduce the detection bandwidth^14^. More recently, the sampling rate of a Brillouin-based sensor has been significantly reduced by means of computational DOFS, an approach that exploits concepts of temporal “ghost imaging”^15^. Another feature of all ΦOTDR schemes is the low power of the backscattered signal coming from the test fibre. This issue is critical since obtaining stronger backscattered signals by increasing the pulse peak power is limited by the onset of modulation instability and other non-linear effects. A time-efficient manner of increasing the signal-to-noise ratio (SNR) is the use of signal coding techniques, where a pulse sequence (differentiated by a particular code) is launched into the fibre within the round-trip time fixed by the fibre length^16–18^. Compared to ΦOTDR techniques based on individual pulses, the increase in the total input power (while maintaining a constant peak power) results in a higher SNR without reducing the measurement rate or spatial resolution. From the sequence of backscattered pulses, the sensing information is retrieved through a decoding algorithm, although at the cost of much larger computational memory and power.

In this paper, we present a novel approach for coherent ΦOTDR sensing based on frequency comb technology. Since the turn of the century, optical frequency combs (OFCs) have revolutionized the field of optical metrology^19^, with applications in astronomy, molecular spectroscopy and optical telecommunications, to cite just a few^20^. In some of those applications, the frequency response of a sample becomes encoded on the set of evenly spaced coherent lines that compose the spectrum of a comb. Each individual line of this probe comb is then resolved by making it interfere with a second comb (acting as a local oscillator, LO), which has a different line spacing. The result of this dual-frequency comb (DFC) scheme is a multi-heterodyne interference that leads to an efficient downconversion of the optical frequencies onto the radio-frequency (RF) domain^21,22^. In this way, an optical bandwidth *BW* composed of thousands of lines can be measured from a very narrow RF comb, with compression factors typically ranging from 10^3^ to 10^5^. In the particular case where OFCs are trains of pulses in the time domain, DFC interference can be viewed as an optical sampling technique^23^.

Among the different technologies for comb generation, electro-optic (EO) modulation of a single continuous-wave laser enables the implementation of DFC systems with off-the-shelf telecommunication components^24,25^. EO comb generators are composed of one or several EO modulators, which can be driven by a variety of RF signals, resulting in OFCs with line separations ranging from 1 MHz to ~10 GHz^25,26^. Some of these driving schemes enable precise control of the spectral phase of a comb and, therefore, of the shape of the periodic signal (often a train of pulses) existing in the time domain^26,27^. In our scheme for coherent ΦOTDR, a test fibre is interrogated by a probe comb with a coding of its spectral phase that avoids the formation of high peak-power pulses. In the time domain, this probe signal is a train of (noise-like) spread waveforms, with a period larger than the fibre round-trip time. The comb signal backscattered by the fibre is then coherently detected in a dual-comb configuration using a second comb with a slightly different period and the same spectral phase coding. Similar to pulse-coding techniques, our method leads to a substantial gain in the SNR when compared to single-pulse ΦOTDR but with straightforward decoding that encompasses only basic processing operations. Additionally, the photo-detected signal required to retrieve the response of the fibre (interferogram) has a duration that is orders of magnitude longer than the period of the probe signal. This time expansion is a consequence of the spectral downconversion that takes place in a DFC system. Thus, spatial resolutions in the centimetre range, which would typically imply GHz optical bandwidths, can be achieved with detection bandwidths in the MHz range or lower. Benefiting from that fact, a dual-comb scheme has recently been reported for quasi-distributed fibre sensing based on a multiplexed Bragg grating array^28^. This approach has been experimentally demonstrated with a low number of sensors (15 gratings) over a short distance (13 m). Despite showing the potential of employing a dual-comb scheme for distance-resolved fibre sensing, this work is based on the generation of optical pulses from a laser cavity, which dramatically limits the attainable SNR and, therefore, the capability to carry out real distributed sensing. In our method, the flexibility offered by EO comb generators makes it possible to tailor the temporal shape of the optical waveforms and, consequently, to tackle the extremely low reflectivity of every elemental section of a long fibre in a ΦOTDR system. Additionally, and unlike a dual-comb system based on a laser cavity, the basic parameters of our sensor can be adapted to the target application by electronically reconfiguring the comb generators.

## Results

### Conceptual design

**Fig. 1:**
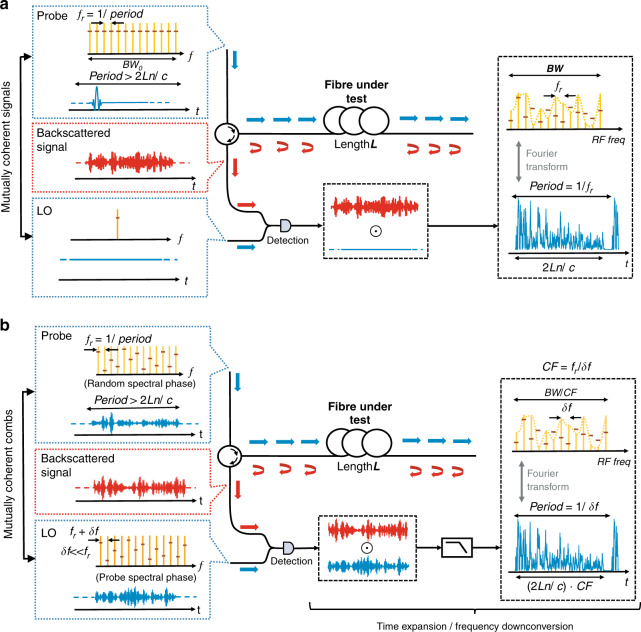
Concept of time-expanded ΦOTDR. **a** An example of a traditional coherent detection-based ΦOTDR scheme. A train of optical probe pulses (with a comb-like spectrum composed of in-phase lines) is launched into the fibre under test. The backscattered light is beaten with a continuous-wave local oscillator (LO) and photo-detected. Both the amplitude and phase of the electromagnetic field are acquired over a bandwidth *BW* identical to that of the launched probe. **b** Proposed time-expanded ΦOTDR scheme. A periodic probe signal is launched into the fibre under test. Its spectrum is a random phase-modulated optical comb. The backscattered light is beaten with an LO that is a comb with the same number of lines and identical phase modulation as those of the probe comb but with a line spacing difference *δf*. After photo-detection, a low-pass filter passes the comb generated by the interference of the lines of the probe with the neighbouring lines of the LO. Both the amplitude and phase of the electromagnetic field are acquired over a compressed bandwidth *BW/CF*, where *CF* is the ratio between the probe line spacing and *δf*. This produces a trace temporally expanded by a factor *CF*. In the detection stage of both figures (central dashed boxes), the signals that are involved in the product (denoted by the symbol ʘ) are electromagnetic fields

A conceptual diagram of our approach and its differences relative to a conventional phase-measuring ΦOTDR system is presented in Fig. 1. In a phase-measuring ΦOTDR, a train of highly coherent optical pulses is injected into a fibre under test (FUT), as shown in Fig. 1a. This probe signal can be, for instance, a train of transform-limited pulses generated by a frequency comb with a flat spectral phase. When the probe signal propagates through the FUT, the randomly reflected fields along the fibre result in a backscattered signal, *e*_*bs*_*(t)*, whose statistical properties are described by a mathematical formalism similar to that of speckle phenomena^29^. This signal can be expressed as the convolution of the input field *e*_1_*(t)* and the impulse response *b(t)* of the FUT, $e_{bs}(t)=e_{1}(t)\ast {b_t}$. In the frequency domain, the backscattered signal is hence *E*_*bs*_(*f*) = *E*_1_(*f*)*B*(*f*), where *E*_*bs*_(*f*), *E*_1_(*f*) and *B*(*f*) are the Fourier transforms of the corresponding time functions^13^. The sensing mechanism relies on the fact that external perturbations, such as strain and temperature, locally change the medium refractive index, producing a significant variation in the phase of *b*(*t*) and, hence, of *e*_*bs*_(*t*). In a coherent detection scheme, the backscattered field *e*_*bs*_(*t*) is mixed with a single-frequency LO, *e*_*LO*_(*t*). From the resulting interference signal, given by the product of *e*_*bs*_(*t*) and $e^{\ast}_{LO}(t)$ (the complex conjugate of the LO field), the amplitude and phase of *e*_*bs*_(*t*) can be retrieved (see Supplementary Material, Eq. S13). In the phase recovery process, the backscattering field and the impulse response can be written in terms of the distance *z* along the fibre using the relation *z* = *ct*/2*n*, where *c* is the speed of light in a vacuum and *n* is the refractive index of the fibre. Thus, the magnitude of an external perturbation is accurately measured along the sensing range through the backscattered signal.

The above conventional scheme for ΦOTDR imposes certain conditions on the sensing system. The spatial resolution of the sensor is set by the temporal width of the probe pulses. This resolution is, therefore, ultimately limited by the optical bandwidth *BW* of the probe signal, which fixes the detection bandwidth (see Fig. 1a). Hence, typical spatial resolutions of a few centimetres require high-speed photo-detection (in the GHz range) with a subsequent increase in the sensing system cost. On the other hand, the SNR of the detected trace is limited, as in any other OTDR configuration, by the energy of the probe signal that can be launched to the system. However, the maximum pulse peak power is constrained by the onset of non-linear effects in the fibre. As mentioned before, several solutions have been proposed to increase the SNR of ΦOTDR, specifically those based on the application of certain probe coding techniques^16–18^. However, these methods decode the sensing information through sophisticated (most often off-line) algorithms that entail a high computational effort.

To tackle the above limitations on the sensing performance, we propose the ΦOTDR scheme sketched in Fig. 1b. The probe signal is a frequency comb generated by an EO modulation scheme, which allows us to select a particular code for the spectral phase to control the peak power of the probe signal, something previously reported for an ultrafine EO comb working in a self-heterodyne scheme^26^. This code consists of allocating a random spectral phase (with a uniform distribution between −*π* and *π*) to each line of the comb^27^ (see Supplementary material, Section S2). In the time domain, it corresponds to a train of repetitive (noise-like) waveforms, with a period given by the inverse of the frequency spacing of the comb, 1/*f*_*r*_. By the use of the random-walk formalism, the magnitude and phase of the resulting temporal signal are demonstrated to follow, respectively, a Rayleigh and a uniform distribution^29^. Hence, the phase code prevents the formation of high peak-power pulse bursts, as occurs when the comb lines are in phase. Indeed, the comb energy is spread along the entire signal period, making it possible to increase the total energy launched into the fibre without the onset of non-linear effects (either in the fibre or owing to the saturation of electro-optical components along the setup). Experimentally, for a sufficiently high number of lines (*N*), the peak-power reduction factor due to the applied spectral phase coding scales linearly with *N*.

As in a conventional sensing scheme, the impulse response of the FUT is encoded onto the backscattered signal. This signal is then coherently detected using a DFC configuration, as shown in Fig. 1b. In that configuration, the LO is a second optical comb, *e*_2_(*t*), with the same spectral phase as that of the probe but with a slightly different line spacing, $f_r + \delta f{\mathrm{ }}\left( {\delta f < < \,f_r} \right)$. As a consequence, the detection turns into a parallel multi-heterodyne process, which can be viewed as the interference of the back-reflected comb with a set of frequency-shifted (coherent) local oscillators. To explain the particularities of our approach, we begin to consider the interference of the two frequency combs in the absence of the FUT. The photo-detected voltage can be written, in a very general manner, as $v\left( t \right) \propto \left[ {e_1\left( t \right)e_2^ \ast \left( t \right)} \right] \ast h_d\left( t \right)$, where $e^{\ast}_{2}(t)$ is the LO complex-conjugated field, *h*_*d*_(*t*) is the impulse response of the detection system (including any hardware or software filtering) and $\ast$ denotes convolution. The spectrum of the detected signal *V*(*f*), after proper low-pass filtering to isolate the interference of neighbouring lines of both optical combs, is demonstrated to be an RF comb composed of a set of teeth located at discrete frequencies $f_q = q\delta f$, with *q* being an integer. In this way, the spectrum of the measured signal results is scaled (when compared to its optical version) by a compression factor $CF = f_r/\delta f$^22^. The complex amplitude of a particular RF tooth is given by $A_{1q}A_{2q}^{\,}\exp \left[ {j(\varphi _{1q} - \varphi _{2q})} \right]$, where *A*_*iq*_ and $\varphi _{iq}$, *i* = (1,2), are the amplitudes and phases of the optical lines of the probe and LO combs (see Section S1 in the Supplementary Material and the classic derivation of ref. ^30^). As explained before, the phase coding of both combs is chosen to be identical; thus, their relative phase is fully cancelled (aside from an irrelevant global phase). Considering $A_{1q} = kA_{2q}$ $\forall q$, where *k* is a constant, the retrieved RF comb is ideally just a flat spectrum. In other words, although the interfering signals are noise-like waveforms due to their random spectral phases, the measured RF signal is a sequence of well-defined electrical pulses (see Eq. S8). If now a test fibre is inserted into this dual-comb scheme, the LO becomes mixed with the backscattered field *e*_*bs*_(*t*). After low-pass filtering, the amplitude and phase of *e*_*bs*_(*t*) can then be directly decoded from the interference signal with no need for any subsequent digital decoding. In this process, the cancellation of the relative phase of the designed combs plays a fundamental role (see Eqs. S9–S18).

Alternatively, dual-comb interference can be understood in the time domain. The interference signal $e_{bs}\left( t \right)e_2^ \ast \left( t \right)$, conveniently discretized, can be expressed, after some mathematical development, as a series of concatenated electric-field cross-correlations (interferograms)^30^. This result simply arises from the fact that there is a difference *δf* between the repetition rates of the interfering time signals. Due to this frequency offset, the overlap between successive periods of the LO and the backscattered signal is time shifted by an effective time step equal to the difference in the repetition periods of both signals. As a consequence, the LO slowly “walks through” an entire optical period of the backscattered signal (*T*_*r*_ = 1/*f*_*r*_). The result is a discrete cross-correlation between the involved fields that allows us to measure the amplitude and phase of *e*_*bs*_(*t*) along the length of the FUT $\left( {L \,<\, T_rc/2n} \right)$. The time extent of this cross-correlation sets the minimum acquisition time and is demonstrated to be $T_{ac} = 1/\delta f$^22,30^. Therefore, each interferogram is measured on an expanded time scale, characterized by an expansion factor $T_{ac}/T_r$, which is equal to the compression factor $CF = f_r/\delta f$ that scales down the optical bandwidth in the frequency domain. Thus, in the time picture, the interrogation of the FUT can be seen as a real-time decoding process performed in the heterodyning itself, where the coding gain is actually adjustable through the repetition-rate difference *δf*.

### Time expansion

**Fig. 2:**
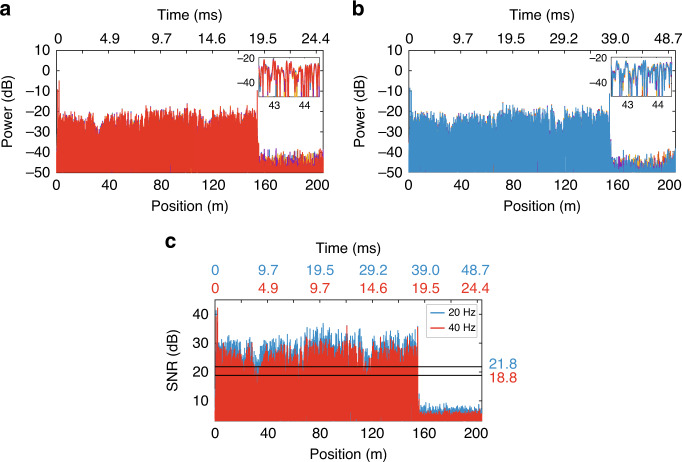
Illustration of the time expansion. Magnitude of the traces detected using **a** a 40-Hz offset and **b** a 20-Hz offset. Insets show the details of several consecutive acquired traces, showing good repeatability. **c** SNR comparison of both measurements, showing a 3dB average increase for the smaller offset, as expected from the reduced acquisition bandwidth. These results show that the proposed method can expand the temporal duration of the trace, leading to a proportional increase in the SNR but with a loss in the maximum measurable acoustic bandwidth

In our first experimental demonstration, the probe and LO combs are configured to have a bandwidth of 2.5 GHz (~4 cm nominal spatial resolution) and a frequency spacing (*f*_*r*_) of 500 kHz (a maximum measurable distance of ~205 m, considering a fibre refractive index of 1.45). This configuration implies 5,000 lines per comb or, equivalently, 5,000 independent measurement points along the fibre. The repetition-rate offset between the two combs (*δf*) can be tuned within a few tens of Hz to produce different time-expansion levels on the optical trace. Figure 2a, b shows the traces acquired when *δf* is 40 and 20 Hz, respectively. The oscilloscope acquisition time is shown at the top of each figure. In the first case (*δf* *=* 40 Hz), the expected time expansion is $CF = f_r/\delta f =\,12,500$. For a sensing length of 154 m, this expected value means that the signal from the fibre is expanded from ~1.5 μs to ~18.7 ms, which is in good agreement with the obtained result. In the second case (*δf* *=* 20 Hz), this duration is doubled, as expected. The insets in these two figures show the details of several consecutive traces acquired with the same settings, demonstrating good repeatability. Figure 2c shows how the SNR changes when the time expansion is modified. As shown, the average trace SNR is 3 dB larger for 20 Hz, the lowest offset. This outcome is in line with the expected result: the signal power is maintained, while the noise variance is reduced to half due to the decrease in the signal bandwidth. Note that this assumption is valid considering only additive, broadband noise sources.

### Sensing results

**Fig. 3:**
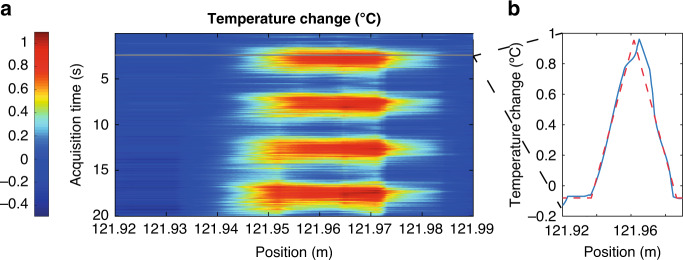
Temperature sensing by the time-expanded ΦOTDR. **a** Dynamic temperature map around the perturbed area. **b** Experimentally obtained temperature profile around the maximum (blue line) and expected (dashed red line) profiles. The latter was obtained by assuming a constant temperature profile along the hotspot; thus, the square-shaped profile of the hotspot is convolved with the 2 cm resolution window given by the gauge length. This results in a triangular shape for the measured temperature profile

To test the performance of our time-expanded scheme, we carry out temperature sensing with the FUT used in Fig. 2 (i.e. over a maximum length of 200 m). For this purpose, we create an ~2 cm hotspot at *z* = 122 m using a pair of metallic wires embedded in the fibre cable (see Materials and Methods for details). The temperature in this hotspot is modulated using a switched current with a 50% duty cycle and a period of 5 s. After a few minutes of temperature stabilization, a sequence of consecutive traces is measured for 20 s at a rate of 20 Hz and saved for off-line processing. The probe and LO comb parameters are now set to *N* = 10,000 lines, *f*_*r*_ = 500 kHz and *δf* = 20 Hz. With the above parameters, the probe bandwidth is 5 GHz, and the nominal spatial resolution is 2 cm. The average power sent to the fibre is approximately 9 dBm, while the peak power is ~14 dB higher (23 dBm), still below the threshold for modulation instability for this fibre length. Previously, for the photodetector, the probe signal and LO were adjusted via variable attenuators to obtain a similar average power of ~−13.8 dBm. The local phase of the backscattered field is recovered simply by filtering one of the two first-Nyquist-zone sidebands of the photo-detected signal (either positive or negative frequencies) and transforming it back to the time domain to recover a complex-valued trace. The phase changes at each position of this complex trace provide the basis for the determination of temperature changes along the fibre, as explained in Section S4 of the Supplementary material. Figure 3a shows the measured temperature profile along the fibre, obtained with a gauge length Δ*z* = 2 cm. As can be seen, we retrieve the expected temperature modulation with a perturbation length that matches Δ*z*. Figure 3b shows the longitudinal temperature distribution obtained at a specific instant of the acquisition, along with the expected distribution (dashed line). The average amplitude of the recorded temperature modulation is ~0.96 °C, as estimated from the calibration in a longer fibre section (see Supplementary Material, Section S5).

**Fig. 4:**
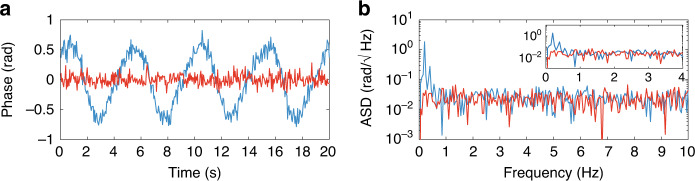
Sensitivity in the temperature sensing experiment. Comparison of the measured phase signal in the hotspot (blue) and at an unperturbed point outside the hotspot (red) in the **a** time domain and **b** frequency domain

The sensitivity of the above sensor is then characterized (Fig. 4) in both the hotspot and unperturbed positions of the fibre. Phase is used as a measurand for simplicity, as it is the variable that most easily allows the system to be compared with other reported ΦOTDR sensors. Note that since temperature/strain perturbations result in a phase change accumulated per metre, the obtained temperature/strain error will depend on the (digitally selected) gauge length even if the same optical parameters are used. First, the noise is measured in all unperturbed positions of the fibre, presenting a median standard deviation of 0.09 rad (corresponding to ~490 *nε* or 55 mK for a 2 cm gauge length—see Eqs. S22 and ref. ^10^) and an average amplitude spectral density (ASD) noise floor of 0.02 ${\mathrm{rad}}{\mathrm{/}}\sqrt {Hz}$. A comparison of the (time domain) noise of an unperturbed point with the signal on the hotspot is presented in Fig. 4a (the DC components of both signals are removed for better visualization). The same comparison is made in the frequency domain (Fig. 4b). Since the signals from the hotspot and from the unperturbed point are affected by white noise with a similar noise floor, the ASD plot shows that the sensor does not present aliasing for the applied perturbations. Finally, the measurements of the developed scheme are compared with those of a calibrated traditional ΦOTDR sensor for a hotspot of 10 m (see Supplementary Material, Section S5). This experiment demonstrates (a) the accuracy/reliability of the developed scheme and (b) its capability of measuring perturbations ranging from a few cm to tens of metres (i.e. hundreds of times larger than the spatial resolution, with hundreds of radians in accumulated phase shift) with the same optical setup, as expected.

### Range expansion

**Fig. 5:**
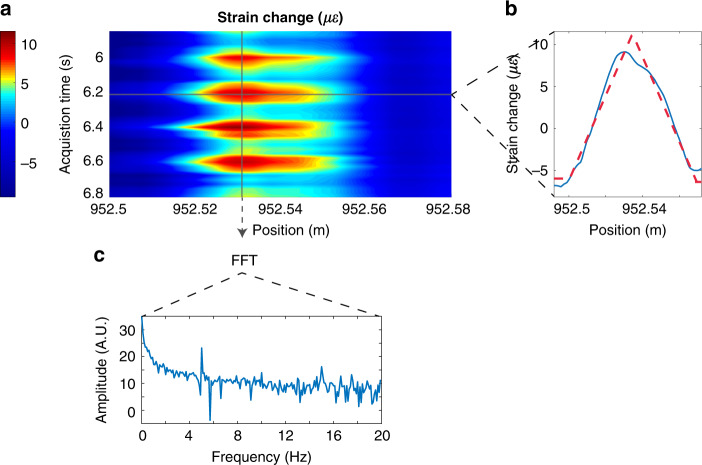
Strain sensing using the QIR-DFC technique over a 1 km fibre. **a** Dynamic stress map around the perturbed area. **b** Stress profile experimentally obtained at a particular instant of time. As in the case of the results obtained for temperature sensing, the observed distribution is the result of convolving the applied stress distribution by a rectangular resolution window, leading to a roughly triangular-shaped profile. **c** Amplitude of the Fourier transform of a vertical profile in **a** located at 952.53 m

The operation range of the proposed sensing scheme is limited due to the need to avoid aliasing in detection. For a given optical bandwidth (set by the spatial resolution), extending the distance range implies increasing the number of probe comb lines to reduce the optical spacing between them (*f*_*r*_). Since the downconverted comb must lie within an RF region that extends from dc to half the optical line spacing (first-Nyquist zone), generating more lines requires adjusting the frequency offset between the combs to a sufficiently low value (to guarantee *Nδf* < *f*_*r*_/2). Because the frequency offset cannot be indefinitely reduced (without dramatically slowing down the measurement process), we cannot increase the density of the comb lines at will. This fact eventually imposes a practical limitation on the length of the FUT. However, it is possible to achieve km-length ranges by implementing a dual-frequency-comb system working in a quasi-integer-ratio mode (which hereafter we will abbreviate as QIR-DFC system)^31^. Some aspects of this technique are detailed in Section S3 of the Supplementary material. In our sensor based on the QIR-DFC scheme, the features of the probe comb (optical bandwidth *BW* and line spacing *f*_*r*_) are determined by the targeted sensing performance, while the LO comb has a similar bandwidth, but its line spacing is a multiple of the probe line spacing plus a frequency offset ($M\cdot f_r + \delta f$). In the time-domain picture, the operation principle of the proposed QIR-DFC system can be understood as a process in which a slow-repetition-rate comb is employed to interrogate the FUT, while a comb that is *M* times faster acts as the LO. This second comb can be visualized as a set of slower interleaved combs; thus, the dual-comb system works with *M*-fold multiplexed channels, leading to the acquisition of *M* offset interferograms simultaneously^31^. Then, by demultiplexing this signal, it is possible to perform dual-comb measurements using a probe with a very small spacing without sacrificing the system speed. In the frequency domain, the interference of the backscattered comb with the LO produces an RF spectrum composed of groups of lines (Nyquist zones) located around $k{\mathrm{ }}f_r$ $\left( {k = 0,1,2,...} \right)$; thus, a downconverted version of the modulated probe can be reconstructed from the first *M* Nyquist zones (see Fig. S1). Thanks to the versatility of our method to generate OFCs, the entire LO comb can be shifted by an amount *δf* with respect to the probe. In that case, the condition to avoid aliasing in detection becomes $\delta f \,<\, M \cdot f_r^2{\mathrm{/}}\left( {2 \cdot BW} \right)$^32^, implying an increase by a factor *M* with respect to the usual condition required by conventional dual-comb spectroscopy^22^. As in the simple time-expansion mode, we control the spectral phase of the combs to ensure that a high average power is sent to the FUT. To illustrate the range expansion provided by the QIR mode, we carry out strain sensing using an FUT with a length of 1003 metres. The setup employed in this experiment is slightly different from that used in the previous section and described in the Supplementary material (Fig. S2). The probe comb in this case is made up of 25,000 lines with a line spacing *f*_*r*_ = 100 kHz, which provides a targeted sensing length of 1 km and a spatial resolution of 4 cm. The LO comb is composed of 500 lines covering the same bandwidth (2.5 GHz); thus, the integer factor *M* = 50. The difference *δf* (which fixes the acoustic sampling) is 40 Hz. In this experiment, we launch an average power of 0.56 dBm into the test fibre, while the peak power reaches 15.12 dBm, a value still below the threshold above which modulation instability occurs. Before the photo-detection stage, the probe average power measured at one output of the optical coupler is −17.4 dBm, while the peak power is ~1 dBm. The LO, in turn, has an average and a peak power similar to those employed in the setup for temperature sensing. The photo-detected signal goes through a low-pass filter with a cut-off frequency of 6 MHz. Examples of measured traces can be observed in Figure S3, which shows an estimated SNR of 9.3 dB and good repeatability. The mechanical perturbation is applied to the FUT over Δ*z* *=* 4 cm at *z* = 952.5 m. This section is stressed under the effect of a shaker, driven by a sinusoidal signal with a frequency of 5 Hz. By applying the QIR reconstruction algorithm (see Section S3 in the Supplementary material), the strain Δ*ε* induced by the shaker is measured (Eq. S23 and ref. ^10^), as summarized in Fig. 5. A dynamic stress map of the perturbed area is shown in Fig. 5a. The maximum strain variation is 14 *με*, and the sensitivity of our system is 350 *nε*, which corresponds to a standard phase deviation of 0.13 rad. Figure 5b shows a longitudinal stress profile acquired at a particular instant of time (6.225 s). The frequency analysis of a vertical profile located at 952.53 m over an acquisition time of 10 s is plotted in Fig. 5c. Two peaks can be observed in the Fourier transform amplitude of that profile at 5 and 15 Hz (first and third harmonic, respectively). The peak at 15 Hz is attributed to the nonlinearity of the actuator. This result confirms the capability of our QIR-DFC scheme to perform dynamic sensing measurements over 1 km.

## Discussion

In summary, we have developed an entirely new concept for arbitrarily expanding the temporal trace in coherent ΦOTDR sensing systems. We have improved by orders of magnitude the number of sensing points (25,000) and the ranging distance (1 km) when compared to the results experimentally demonstrated for quasi-distributed sensing in ref. ^28^. This time expansion is similar to that achieved by linear optical sampling (LOS) techniques^33,34^. In standard LOS, a periodic test signal (usually featuring a high-repetition rate) is sampled by a train of short pulses with longer periods, which acts as a gate function. Due to the repetition-rate difference, the sampling of the test signal by consecutive short pulses requires many gating periods. Thus, relatively slow detectors and low-bandwidth electronics enable the measurement of high-repetition-rate signals. A dual-comb system that generates trains of optical pulses can be utilized to implement coherent linear optical sampling with high time expansion (*CF* = 10^4^)^23^. However, if applied to ΦOTDR, this scheme would suffer from the limitations of the SNR derived from the use of optical pulses. This is the reason why shaping the temporal profile of the signals (through control of the spectral phases of the combs) massively improves the SNR while maintaining the linear optical sampling time expansion.

Our time-expanded approach overcomes one of the long-standing trade-offs existing in ΦOTDR systems, namely, the one existing between the spatial resolution and detection bandwidth, allowing the achievement of centimetre resolution with MHz RF bandwidths. However, to position the technology, its performance is compared here (see Table 1) with that of state-of-the-art distributed sensors that can operate in a similar spatial resolution range (i.e. centimetres) and acoustic sampling range (tens of hertz), namely, different types of coded ΦOTDR and dynamic optical frequency-domain reflectometry (OFDR). Traditional single-shot ΦOTDR is unable to reach cm resolutions under normal operation, as this would require the use of sub-ns pulses with low energy, which greatly decreases the measurement SNR. Thus, different methods to increase the probe pulse duty cycle without degrading the resolution have been proposed, in all cases adding significant complexity to the measurement process. For example, ΦOTDR using swept frequency pulses and matched filtering techniques has been demonstrated to allow for spatial resolutions proportional to the probe spectral content (and corresponding detection bandwidth), reaching cm spatial resolutions under good SNR conditions^35,36^. Spatial resolutions down to 2.5 cm have also been demonstrated in coded ΦOTDR^18^. Note that in all these approaches, GHz bandwidth detection and digitalization are required (often with multiple photodetectors), which drastically increases the cost, complexity and computational needs of the system. The main advantage of this set of techniques is the large attainable range with sub-meter resolution (e.g. 50 km with a 0.3 m resolution^37^, 10 km with a 0.8 m resolution^38^ and 10 km with a 0.92 m resolution^39^, all with kHz acoustic samplings).

**Table 1:** Comparison of dynamic distributed fibre sensing techniques operating in the cm spatial resolution region.

Type of interrogation	Spatial resolution	Sensitivity	Fibre length	Acoustic sampling	Detection bandwidth required	Detection type	Probe modulation bandwidth
Pulse compression ΦOTDR^38^	10 cm	No dynamic perturbation measured	58 km	~kHz	~1 GHz	1 PD per pol. axis	1 GHz (external modulation)
Pulse-coding ΦOTDR^18^	2.5 cm	~0.1 rad (~0.4*με* @ 2.5 cm)	500 m	122 kHz	~4 GHz	2 PD per pol. axis	4 GHz (external modulation)
OFDR^42^	20 cm	~0.4 rad (200*με* @ 20 cm)	30 m	50 Hz	~MHz	Multiple detection stages	~5 THz (tuneable laser source)^a^
OFDR^43^	10 cm	~1 rad (~1*με* @ 10 cm)	200 m	300 Hz	~5 MHz	Multiple detection stages	~2.5 THz (tuneable laser source)^a^
OFDR^44^	12 cm	~0.1 rad (~100 *με* @ 10 cm)	950 m	6.25 kHz	~1 GHz	1 PD per pol. axis	8 GHz (external modulation)
TE-ΦOTDR (proposed in this work)	2 cm	0.09 rad (~490 *με* @ 2 cm)	200 m	20 Hz	<1 MHz	1 PD per pol. axis	5 GHz (DFC scheme)
TE-ΦOTDR (QIR mode) (proposed in this work)	4 cm	0.13 rad (~350 *με* @ 4 cm)	1000 m	40 Hz	<6 MHz	1 PD per pol. axis	2.5 GHz (QIR-DFC scheme)

^a^Measurements demonstrated only within the duration of one sweep period of the tuneable laser source (~1 s)

In OFDR systems, the frequency of a laser is swept, and a heterodyne detection scheme is used to beat the light from the laser with the one backscattered by the FUT. As a result of this process, each beat frequency is directly related to a particular location along the fibre, as long as the frequency sweep is perfectly linear. In principle, the spatial resolution is given by the inverse of the frequency range covered by the sweep. For this reason, while pure OFDR schemes can be made to operate with performances similar to that of ΦOTDR, they are usually tailored to provide high spatial resolutions over shorter fibres and smaller acoustic bandwidths, thereby requiring a lower detection bandwidth than that of comparable ΦOTDRs^40–43^. It must be noted, however, that achieving a high spatial resolution requires minimizing the degrading effect of sweep nonlinearities. Generally, this implies including in the OFDR setup an auxiliary interferometer (to measure the instantaneous frequency) and combining it with interpolation algorithms to correct the frequency sampling^43^. This auxiliary interferometer must be tailored to the targeted measurement distance, with the subsequent limitation of changing the sensing range, and the correcting process demands data acquisition hardware with large memory, as well as intensive processing. Additionally, a high SNR is usually assured by integrating the signal across a large number of measured points, thus reducing the attainable spatial resolution or forcing sweeps to be carried out over very broad bandwidths. Recently, OFDR sensors with a resolution of tens of centimetres, acoustic sampling of tens to hundreds of Hz and fibre of tens to hundreds of metres have been demonstrated with detection bandwidths at the MHz scale^42,43^ (see Table 1). However, in all these cases, the laser was required to linearly sweep over tens of nanometres, with specific controls and detection stages aimed at guaranteeing it. In a somewhat intermediate configuration between the two sets of techniques, phase-sensitive OFDR with an externally modulated probe (8 GHz spectral content) demonstrated 12 cm resolution over 950 m and 6.25 kHz acoustic sampling but still required ~1 GHz (and two photodetectors) for detection. The sensor presented a standard deviation of ~0.1 rad (corresponding to ~100 *nε*) after fading suppression. This result implies a performance closer to the one demonstrated here but with a much higher detection bandwidth^44^. The two experimental demonstrations developed in this work showed spatial resolutions <4 cm, acoustic samplings up to 40 Hz and a range up to 1 km. In both experiments, the amplitude spectral density noise floor was ~0.02 ${\mathrm{rad}}{\mathrm{/}}\sqrt {Hz}$. One of the most salient features of the developed technique is that the detection bandwidth is reduced by orders of magnitude in comparison with the probe bandwidth (a few GHz in our demonstrations, corresponding to the spatial resolutions achieved) while still showing a pure time-domain readout. On the other hand, the main sacrifice of the technology is achievable acoustic sampling, which is also reduced by orders of magnitude in comparison with a typical time-domain system. Nevertheless, for a large number of applications (e.g. structure health monitoring in the aerospace, marine or automotive sectors^45^, diagnostics in industrial manufacturing applications^46^, and borehole monitoring in seismology and reservoir surveillance^47^), the acoustic sampling demonstrated here (40 Hz) may provide real-time information. Overall, compared to other schemes (see Table 1), the time-expanded system presented here offers much less measurement complexity than that of comparable ΦOTDR/OFDR systems (only one low-bandwidth photoreceiver is needed) while still presenting comparatively high spatial resolution and acceptable acoustic sampling for most applications. Note that the configuration presented in this paper does not avoid the usual problem of fading in phase-OTDR configurations^1^. However, the time-expansion method is definitely compatible with many of the fading suppression strategies already presented in the literature, such as multi-wavelength^48^ or multi-carrier probing^49^. On the other hand, although laboratory arbitrary waveform generators (AWGs) with a high analog bandwidth are expensive instruments, industrialized arbitrary waveform generation can be developed, at a relatively low cost and even at gigahertz speeds, by using field-programmable gate arrays (FPGAs) and digital-to-analog conversion^50^. Therefore, with suitable engineering of the comb signal generation stage, the potential cost of the system could be considerably reduced in applications not requiring particularly high acoustic samplings.

## Materials and methods

**Fig. 6:**
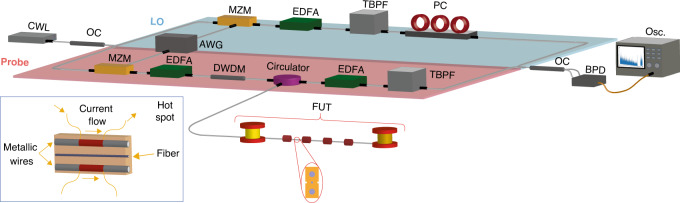
Experimental setup for time-expanded ΦOTDR. OC: optical coupler. Osc.: oscilloscope. The remaining acronyms are explained in the text. The inset in the lower-left corner is a sketch of the employed hotspot for the temperature sensing experiment

The setup implemented for the time-expanded ΦOTDR sensor is shown in Fig. 6. Here, we provide details of the system employed for temperature sensing using combs with similar line spacings. We include a description of the sensor based on the QIR-DFC scheme in Section S3 of the Supplementary material. As can be observed in Fig. 6, the light source is a continuous-wave laser (CWL) with an ultranarrow linewidth (NKT Koheras Basik X15, linewidth <0.1 kHz). This laser seeds two comb generators, each composed of an intensity Mach-Zehnder modulator (MZM, Photline-MX-LN-10 for the probe and Oclaro SD20 for the LO) driven by an AWG (2-channel M8195A Keysight, with a 25 GHz analog bandwidth, a 65 GSPS maximum output sample rate, a memory depth of 1.6 GSamples per channel and an 8 bit vertical resolution). The process to generate a flat-topped frequency comb by electro-optic modulation is briefly explained in the Supplementary material (Section S2). Basically, tailored RF signals are first computationally designed and subsequently generated with a single AWG. These signals are real-valued; thus, their spectrum is composed of two symmetric complex-conjugated sidebands centred at *f* = 0 Hz^27^. After the modulation stage, the optical signals are boosted by erbium-doped fibre amplifiers (EDFAs, AOC MEDFA-17–0.5-FC). The probe comb is subsequently filtered by a dense wavelength division multiplexer (DWDM) to reduce the amplified spontaneous emission introduced by the EDFAs. The resulting signal is launched into 154 m of standard G.652 optical fibre. The backscattered signal from this FUT is collected by an optical circulator and then amplified by another EDFA. To ensure a correct downconversion upon detection, one sideband of each comb is suppressed by means of optical tuneable bandpass filters (TBPFs, Yenista XTM-50). The TBPFs also filter out part of the amplified spontaneous emission introduced by the EDFAs. The interference between both combs is detected with the aid of a balanced photodetector with a bandwidth of 100 MHz (BPD, Thorlabs PDB 410 C). The generated electrical signal is externally bandlimited by a low-pass filter with a cut-off frequency of 2.5 MHz and digitized by an oscilloscope (8-bit 13 GHz model Agilent DSO91304A). Since the RF spectrum within the first-Nyquist zone extends over 200 kHz, an acquisition card with a much lower speed may be used instead. Several polarization controllers (PCs) are included in the setup to maximize the interference signal.

To perform temperature sensing, the structure of the employed FUT has two metallic wires embedded into the fibre cabling. These wires run parallel to the optical fibre, all along the distance. By flowing an electrical current through the wires, Joule heating can induce a temperature change in the fibre (see Fig. 6). To create localized heating in this case, access to the wires is created with a separation of ~2 cm, and a current is flown through the 2 cm section. The current is modulated to create periodic heating-cooling cycles once a stable temperature is reached. Since the wire section is basically homogeneous along the distance, the resistance per unit length can be assumed to be constant. Thus, for a given current flow, the power dissipation (and hence the temperature) can also be assumed to be constant along the distance over which the current is flown. The temperature modulation induced in the fibre as a function of the current is calibrated using chirped-pulse phase-sensitive reflectometry^12^ (see Section S5 in the Supplementary Material).

## Supplementary information

Supplementary Information for Time-expanded phase-sensitive optical time-domain reflectometry

## References

[1] Hartog, A. H. *An Introduction to Distributed Optical Fibre Sensors* (CRC Press, 2017).

[2] Bao XY, Chen L (2012). Recent progress in distributed fiber optic sensors. Sensors.

[3] Denisov A, Soto MA, Thévenaz L (2016). Going beyond 1000000 resolved points in a Brillouin distributed fiber sensor: theoretical analysis and experimental demonstration. Light. Sci. Appl..

[4] Juarez JC (2005). Distributed fiber-optic intrusion sensor system. J. Lightwave Technol..

[5] Juarez JC, Taylor HF (2007). Field test of a distributed fiber-optic intrusion sensor system for long perimeters. Appl. Opt..

[6] Martins HF (2014). Phase-sensitive optical time domain reflectometer assisted by first-order raman amplification for distributed vibration sensing over >100 km. J. Lightwave Technol..

[7] Wang ZN (2014). Phase-sensitive optical time-domain reflectometry with Brillouin amplification. Opt. Lett..

[8] Lu YL (2010). Distributed vibration sensor based on coherent detection of phase-OTDR. J. Lightwave Technol..

[9] Tu GJ (2015). The Development of an Φ-OTDR system for quantitative vibration measurement. IEEE Photonics Technol. Lett..

[10] Koyamada Y (2009). Fiber-optic distributed strain and temperature sensing with very high measurand resolution over long range using coherent OTDR. J. Lightwave Technol..

[11] Zhou L (2015). Distributed strain and vibration sensing system based on phase-sensitive OTDR. IEEE Photonics Technol. Lett..

[12] Pastor-Graells J (2016). Single-shot distributed temperature and strain tracking using direct detection phase-sensitive OTDR with chirped pulses. Opt. Express.

[13] Pastor-Graells J (2017). SNR enhancement in high-resolution phase-sensitive OTDR systems using chirped pulse amplification concepts. Opt. Lett..

[14] Foaleng-Mafang, S., Beugnot, J. C. & Thevenaz, L. Optical sampling technique applied to high resolution distributed fibre sensors. in *Proc. SPIE 7503, 20th International Conference on Optical Fibre Sensors*. SPIE, 2009.

[15] Zhou DP (2019). Computational distributed fiber-optic sensing. Opt. Express.

[16] Jones MD (1993). Using simplex codes to improve OTDR sensitivity. IEEE Photonics Technol. Lett..

[17] Lee D (2006). Optimization of SNR improvement in the noncoherent OTDR based on simplex codes. J. Lightwave Technol..

[18] Martins HF (2016). Real time dynamic strain monitoring of optical links using the backreflection of live PSK data. Opt. Express.

[19] Udem T, Holzwarth R, Hänsch TW (2002). Optical frequency metrology. Nature.

[20] Newbury NR (2011). Searching for applications with a fine-tooth comb. Nat. Photonics.

[21] Coddington I, Swann WC, Newbury NR (2008). Coherent multiheterodyne spectroscopy using stabilized optical frequency combs. Phys. Rev. Lett..

[22] Coddington I, Newbury N, Swann W (2016). Dual-comb spectroscopy. Optica.

[23] Coddington I, Swann WC, Newbury NR (2009). Coherent linear optical sampling at 15 bits of resolution. Opt. Lett..

[24] Long DA (2014). Multiheterodyne spectroscopy with optical frequency combs generated from a continuous-wave laser. Opt. Lett..

[25] Durán V, Tainta S, Torres-Company V (2015). Ultrafast electrooptic dual-comb interferometry. Opt. Express.

[26] Bao Y (2015). A digitally generated ultrafine optical frequency comb for spectral measurements with 0.01-pm resolution and 0.7-µs response time. Light. Sci. Appl..

[27] Soriano-Amat M (2020). Common-path dual-comb spectroscopy using a single electro-optic modulator. J. Lightwave Technol..

[28] Zhao X (2020). Dynamic quasi-distributed ultraweak fiber Bragg grating array sensing enabled by depth-resolved dual-comb spectroscopy. IEEE Trans. Instrum. Meas..

[29] Wójcik, A. K. *Signal statistics of phase dependent optical time domain reflectometry*. PhD thesis, Texas A&M University, 2006.

[30] Newbury NR, Coddington I, Swann W (2010). Sensitivity of coherent dual-comb spectroscopy. Opt. Express.

[31] Hébert NB (2014). Coherent dual-comb interferometry with quasi-integer-ratio repetition rates. Opt. Express.

[32] Klee A (2013). Characterization of semiconductor-based optical frequency comb sources using generalized multiheterodyne detection. IEEE J. Sel. Top. Quantum Electron..

[33] Schmidt-Langhorst C, Weber HG (2005). Optical sampling techniques. J. Optical Fiber Commun. Rep..

[34] Dorrer C (2003). Linear optical sampling. IEEE Photonics Technol. Lett..

[35] Zou WW (2015). Optical pulse compression reflectometry: proposal and proof-of-concept experiment. Opt. Express.

[36] Yu L, Zou WW, Chen JP (2016). Optical pulse compression reflectometry based on double sideband modulation. IEEE Photonics Technol. Lett..

[37] Mompó JJ (2018). Sidelobe apodization in optical pulse compression reflectometry for fiber optic distributed acoustic sensing. Opt. Lett..

[38] Chen D, Liu QW, He ZY (2018). High-fidelity distributed fiber-optic acoustic sensor with fading noise suppressed and sub-meter spatial resolution. Opt. Express.

[39] Wang ZN (2019). Distributed acoustic sensing based on pulse-coding phase-sensitive OTDR. IEEE Internet Things J..

[40] Ding ZY (2018). Distributed optical fiber sensors based on optical frequency domain reflectometry: a review. Sensors.

[41] Arbel D, Eyal A (2014). Dynamic optical frequency domain reflectometry. Opt. Express.

[42] Zhou DP, Chen L, Bao XY (2016). Distributed dynamic strain measurement using optical frequency-domain reflectometry. Appl. Opt..

[43] Li J (2017). High spatial resolution distributed fiber strain sensor based on phase-OFDR. Opt. Express.

[44] Li H (2020). High-spatial-resolution fiber-optic distributed acoustic sensor based on Φ-OFDR with enhanced crosstalk suppression. Opt. Lett..

[45] Lally, E. M. et al. Fiber optic shape sensing for monitoring of flexible structures. in *Proc. SPIE 8345, Sensors and Smart Structures Technologies for Civil, Mechanical, and Aerospace Systems 2012*. SPIE, 2012.

[46] Ukil A, Braendle H, Krippner P (2012). Distributed temperature sensing: review of technology and applications. IEEE Sens. J..

[47] Daley TM (2013). Field testing of fiber-optic distributed acoustic sensing (DAS) for subsurface seismic monitoring. Lead. Edge.

[48] Lu, Y. et al. Fading noise reduction in distributed vibration measurements utilizing multi-wavelength based Φ-OTDR. in *Proc. 26th International Conference on Optical Fiber Sensors*. Optical Society of America, 2018.

[49] Zhang JD (2019). 80 km fading free phase-sensitive reflectometry based on multi-carrier NLFM pulse without distributed amplification. J. Lightwave Technol..

[50] Zhang, H. F. et al. High-speed arbitrary waveform generator based on FPGA. in *Proc. 2013 IEEE Nuclear Science Symposium and Medical Imaging Conference*. IEEE, 2013.

