# Supplementary material for: Time-expanded phase-sensitive optical time-domain reflectometry
Source: Light Sci Appl. 2021 Mar 9;10:51. doi: 10.1038/s41377-021-00490-0 (PMC7940432; doi:10.1038/s41377-021-00490-0)
Supplement: Supplementary file 1 — Supplementary Information for Time-expanded phase-sensitive optical time-domain reflectometry [file 41377_2021_490_MOESM1_ESM.docx]

**Supplementary Information for Time-expanded phase-sensitive optical time-domain reflectometry**

Miguel Soriano-Amat^1*^, Hugo F. Martins^2^, Vicente Durán^3^, Luis Costa^1^, Sonia Martin-Lopez^1^, Miguel Gonzalez-Herraez^1^ and María R. Fernández-Ruiz^1^

^1^Departamento de Electrónica, Universidad de Alcalá, Escuela Politécnica Superior, 28805 Madrid, Spain

^2^Instituto de Óptica “Daza de Valdés”, IO-CSIC, C/Serrano 121, 28006 Madrid, Spain

^3^GROC-UJI, Institute of New Imaging Technologies, University Jaume I, 12071 Castellón, Spain

*S1. Analytical model of the time-expanded ΦOTDR*

An ideal frequency comb can be simply described in the frequency domain as a series of Dirac delta functions

[S1]

where $A_{m}$,$\upsilon_{m}$ and are the amplitude, frequency and phase of the $m^{th}$ comb line. Thus, the electric field of an ideal frequency comb, $e\left(t\right)=F^{-1}\left\{E\left(f\right)\right\}$ (with $F^{-1}\left\{\cdot\right\}$ representing the inverse Fourier transform), can be simply written as a sum of complex exponentials:

[S2]

Additionally, the frequency of each comb line can be written in terms of two frequencies: the repetition rate $f_{r}$, which sets the comb spacing, and the frequency of a reference line $\upsilon_{0}$. Here, $\upsilon_{0}$ is considered to be the lowest frequency of a comb composed of $\left(N+1\right)$ lines, so

$\upsilon_{m}=\upsilon_{0}+mf_{r}$ [S3]

where $m$ is the comb line index, with $m=0$ for $\upsilon_{0}$. The total optical bandwidth of the comb spectrum is then $BW=N\cdot f_{r}$. A frequency comb can effectively interrogate a sample of interest at each frequency obeying Eq. S3 and the transfer function of the sample becomes encoded on the amplitude and phase of the comb teeth. The resulting spectral sampling is thus equivalent to the comb’s repetition rate $f_{r}$. The available spectral resolution is determined by the linewidth of each individual tooth (ultimately limited by the linewidth of the laser). Typical readout techniques find it extremely challenging to extract the information encoded on the comb without any loss of resolution. A manner of tackle this problem is to use an alternative readout approach, based on multi-heterodyne detection, which enables an optical-to-radiofrequency (RF) mapping using a very narrow detection bandwidth. The idea is to mix the probe comb with a second comb that has a slightly different repetition rate and acts as a local oscillator (LO). This technique is commonly referred to as *dual-comb spectroscopy* (DCS).  In complex representation (i.e., obviating the complex-conjugated component at negative frequencies), two combs interfering on a photodetector produce a voltage signal of the form

$$V\left(t\right)\propto\left[e_{1}(t)e_{2}^{*}(t)\right]*h_{d}\left(t\right)$$

[S4]

where we have also neglected the terms that do not arise from the interference. The operation indicates convolution. . The indices 1 and 2 refer, respectively, to the first and second comb (the probe and the LO), $m$ and $l$ are comb-line indices, and $h_{d}\left(t\right)$ is the impulse response of the low-pass detection system (e.g., a photodetector with or without an additional RF low-pass filter). By comparison with Eq. S1, the double sum in the last expression indicates that the interference of two optical combs produces a series of RF combs. If $h_{d}\left(t\right)$ is chosen to isolate the lowest-frequency tones below $f_{r}/2$, which are generated by pairs of adjacent optical teeth ($m=l$), we obtain a single RF comb given by:

[S5]

[S6]

The amplitude and phase response of a sample interrogated by the first comb at optical frequencies $\upsilon_{1m}$ are encoded on $A_{1m}$ and , respectively, and appear at RF frequencies $\upsilon_{1m}-\upsilon_{2m}$. From Eq. S3, these frequencies can be written as:

$f_{m}=\upsilon_{1m}-\upsilon_{2m}=\delta f_{0}+m\delta f$, [S7]

where $\delta f$ is the repetition rate difference between the two combs, and we have assumed that the difference $\upsilon_{10}-\upsilon_{20}$ is $\delta f_{0}$, enabling the decoding of information from the first line of the comb. In our implementation, and henceforth in this analytical model, we consider $\delta f_{0}=\delta f$ The detected comb then has a bandwidth of $BW_{RF}=N\delta f$. This implies a reduction with respect to the probe bandwidth that is quantified by a compression factor (CF) defined as $CF=BW_{0}/BW_{RF}=f_{R}/\delta f$.

Ideally, in DCS, the relative amplitudes and phases of the comb lines in the probe and LO are chosen to be constant, i.e.,$A_{1m}/A_{2m}=k_{1}$ and , with $k_{1}$and $k_{2}$being constant values. If all the complex exponentials (or spectral tones) that compose the combs are in phase (, $q$ being a constant value for all $m$), the corresponding time-domain waveforms are trains of pulses with a period of $1/f_{r}$. and a temporal shape given by the inverse Fourier transform of the spectral comb envelope (e.g., sinc-like temporal pulses in the case of a rectangular flat-top comb) . To avoid the formation of optical pulses when interrogating a sample, we assign a random spectral phase (with uniform distribution between $-\pi$ and $\pi$) to each line of the probe comb. The temporal waveform thus acquires a periodic speckle-like shape, in which the energy is distributed along the period $1/f_{r}$. Eluding the formation of high-peak-power optical pulses is beneficial for increasing the signal-to-noise ratio (SNR) of the comb generated in our electro-optical system. Since the total power of one period is quasi-evenly distributed along its duration (following a speckle pattern), the peak power is significantly reduced. Hence, the total signal power can be increased up to reach the saturation of the modulator driver and/or the photodetector. By observing Eq. S5, it is simple to deduce that, by coding the spectral phase of the LO comb using the same random code as applied to the probe comb (i.e., accomplishing ), the RF comb obtained after photodetection can be modeled as

$V_{m=l}(t)\propto\left[\sum_{m=0}^{N} A_{1m}A_{2m}\exp\left[j\left(2\pi\left(\upsilon_{1m}-\upsilon_{2m}\right)t+k_{2}\right)\right]\right]$. [S8]

Equation S8 represents an in-phase spectrally compressed RF comb whose waveform forms electrical pulses. Hence, by using this strategy, the SNR of the optical combs can be significantly increased by means of a proper phase spectral coding, while the detected signal directly provides the impulse response of the sample with no need for further post-processing.

Once the principle of operation of our approach is established, let us model the interrogation of an optical fiber by means of ΦOTDR using DCS. The probe and LO combs are defined as

[S9]

and

, [S10]

where, for the sake of simplicity, we have assumed that both combs have the same amplitude $\left(A_{m}\right)$ and phase .The probe comb is launched into the fiber under test (FUT), which has an impulse response $b(t)$. The backscattered light is the result of the spectral sampling of $b(t)$ by the probe comb

, [S11]

. [S12]

If a single comb is used for the interrogation of the FUT, the impulse response of the fiber $b(t)$ (over the available bandwidth $BW$) can be easily obtained by deconvolving the backscattered electrical field and the probe comb. Let us assume that the backscattered electrical field is acquired by means of heterodyne detection, using a single-tone local oscillator $e_{LO}\left(t\right)=A_{LO}\exp\left[j\left(2\pi\upsilon_{LO}t\right)\right]$ (without loss of generality, we assume ):

. [S13]

The deconvolution process can be simply implemented in the spectral domain as , where $E_{1ds}(f)$ is a version of $E_{1}(f)$ downshifted by $\upsilon_{LO}$. The resulting estimation of the FUT impulse response is

, [S14]

where

$\upsilon_{1m}^{d}=\upsilon_{0}-\upsilon_{LO}+mf_{r}=\upsilon_{\alpha}+mf_{r}$. [S15]

is reconstructed with the spatial resolution and the spectral sampling provided by the generated probe comb. If DCS is employed, instead of acquiring the backscattered electrical field by mixing it with a single-tone local oscillator, $e_{bs}\left(t\right)$is mixed with a LO comb, so

$V_{bs}^{DCS}\left(t\right)\propto\sum_{m=0}^{N} A_{m}^{2}\left|B\left(\upsilon_{1m}\right)\right|\exp\left[j\left(2\pi\left(\upsilon_{1m}-\upsilon_{2m}\right)t+\left(\angle B\left(\upsilon_{1m}\right)+k_{2}\right)\right)\right]$,     [S16]

where the constant phase $k_{2}$ is omitted with no loss of generality. As it can be seen in Eq. S16, the phase of the probe comb is automatically demodulated by the LO comb. The impulse response can be obtained from the above signal, provided that each complex exponential of $V_{bs}^{DCS}\left(t\right)$ is normalized by the corresponding $A_{m}^{2}$. In the case where all the complex exponentials have the same amplitude ($A_{m}=p$, $p$ being a constant value for all $m$), . The frequency of the comb lines is given by Eq. S7. Let us compare the expression obtained by substituting Eq. S15 into Eq. S14 with that derived when substituting Eq. S7 in :

                              [S17]

and

.                        [S18]

By inspection of these two expressions, and neglecting the frequency value of the mode $m=0$ , it can be inferred that is a temporally-expanded version of , where

.                                                [S19]

# *S2. Description of the comb generation*

The basic idea to generate two electro-optic optical frequency combs (EO-OFCs) is to send a tailored train of electrical waveforms to each modulator. In order to ensure the synchronization between the RF signals that are sent to the modulators, we use a single arbitrary waveform generator (AWG). Both signals are previously designed off-line through a process that involves, in a first stage, the numerical construction on a computer of two frequency combs, each one with a line spacing given by $f_{r}$ and $f_{r}+\delta f$, respectively. The parameters of these two combs (i.e. total number of lines, optical bandwidth and frequency offset) are subject to a series of constraints described in detail in [1]. A random phase is allocated to each spectral line of the combs in order to avoid the formation of high peak-power pulses. This random phase is uniformly distributed in the range $\left[-\pi,\pi\right]$ . Once the individual combs are numerically built, they are transformed into time-domain signals through an inverse Fourier transformation. Note that, in order to recover a real-valued signal at the end of the design process, the spectrum of the comb must be conjugated symmetrically around $f=0$ Hz. The resulting time-domain waveforms are then loaded into the AWG, and the generated electrical outputs are boosted by RF amplifiers (Wenteq ABP-1200-01-1825 and WJ SA1137-2A, not shown in Figure 6 of the main text) and then fed into the EO modulators. The two combs are inherently synchronized since they are generated from the same laser and the modulation signals are originated from a single synchronization clock in the AWG. Actually, the temporal waveforms of both combs are essentially the same, except for the fact that one of them (the signal yielding the slightly broader comb) shows a slightly shorter period than the other. This is expectable as the two combs can be related through a simple spectral stretching operation.

# *S3. Distributed sensing by means of a dual-comb system working in a quasi-integer ratio mode*

The improvement in the acoustic sampling provided by the quasi-integer-ratio (QIR) mode can be easily understood from the frequency domain picture of the detection process, as is shown in Figure S1. The probe is designed to have an optical bandwidth (BW) and a line spacing $\left(f_{r}\right)$ adapted to the targeted resolution and sensing range, with a total number of lines $N=BW/f_{r}$. The local oscillator, on its turn, is designed to have much less lines but covering the same bandwidth. In particular, the LO line spacing is set to be a multiple of the probe spacing plus a small offset, $M\cdot f_{r}+\delta f$  (with $\delta f<<f_{r}$). Figure S1a illustrates a simple example of this dual-comb configuration with $M=3$. When both OFCs interfere, every line of the probe beats with every line of the LO, originating a RF spectrum composed of groups of lines located around $k\cdot f_{r}$ $\left(k=0,1,2,...\right)$. The versatility of our method to generate OFCs allows us to shift the entire LO comb by an amount $\delta f$respect to the probe. In that case, the condition to avoid aliasing [2], assuming a large number of probe lines, is given by $\delta f<M\cdot f_{r}^{2}/(2\cdot BW)$. This inequality implies an increase by a factor $M$ respect to the usual condition required by conventional DCS [3]. In this way, the acquisition rate (fixed by $\delta f$) can be sped up, although at the cost of increasing the RF bandwidth. For instance, if $f_{r}=\text{ } 100$ kHz, $BW=2.5$GHz and $M=50$, then $\delta f<\text{ } 100$ Hz. In this example, resolving 25 000 probe lines requires a minimum RF bandwidth of just a few megahertz (very approximately, half the LO line spacing [2]). Another relevant feature of the QIR-dual-frequency comb scheme is that the recovery of the optical lines in the frequency domain is not unique, so a variety of algorithms can be implemented [2], [4], as is schematically illustrated in Figure S1b. Once the algorithm is chosen, and after rearranging the RF lines, the spectral information encoded on the comb probe can be retrieved, as is observed in Figure S1c.

In the time-domain, the spectral re-arrangement of the lines can be simply performed using a re-ordering of the measured interferogram samples. Actually, the resulting temporal waveform in detection can be seen as a series of delayed time-domain interferograms, which contain spatially under-sampled measurements of the fiber trace (this picture is comprehensively described in [5]). By a suitable interleaving algorithm of the time-domain samples, the complete fiber trace can be directly reconstructed. Note that, for a proper operation of this scheme, the phase of each line of the local oscillator must match that of the $M$ neighbouring lines of the probe comb. If this is not ensured, each line of the down-converted spectrum would incorporate an arbitrary fixed phase that would have to be corrected in the frequency domain by means of a calibration measurement.


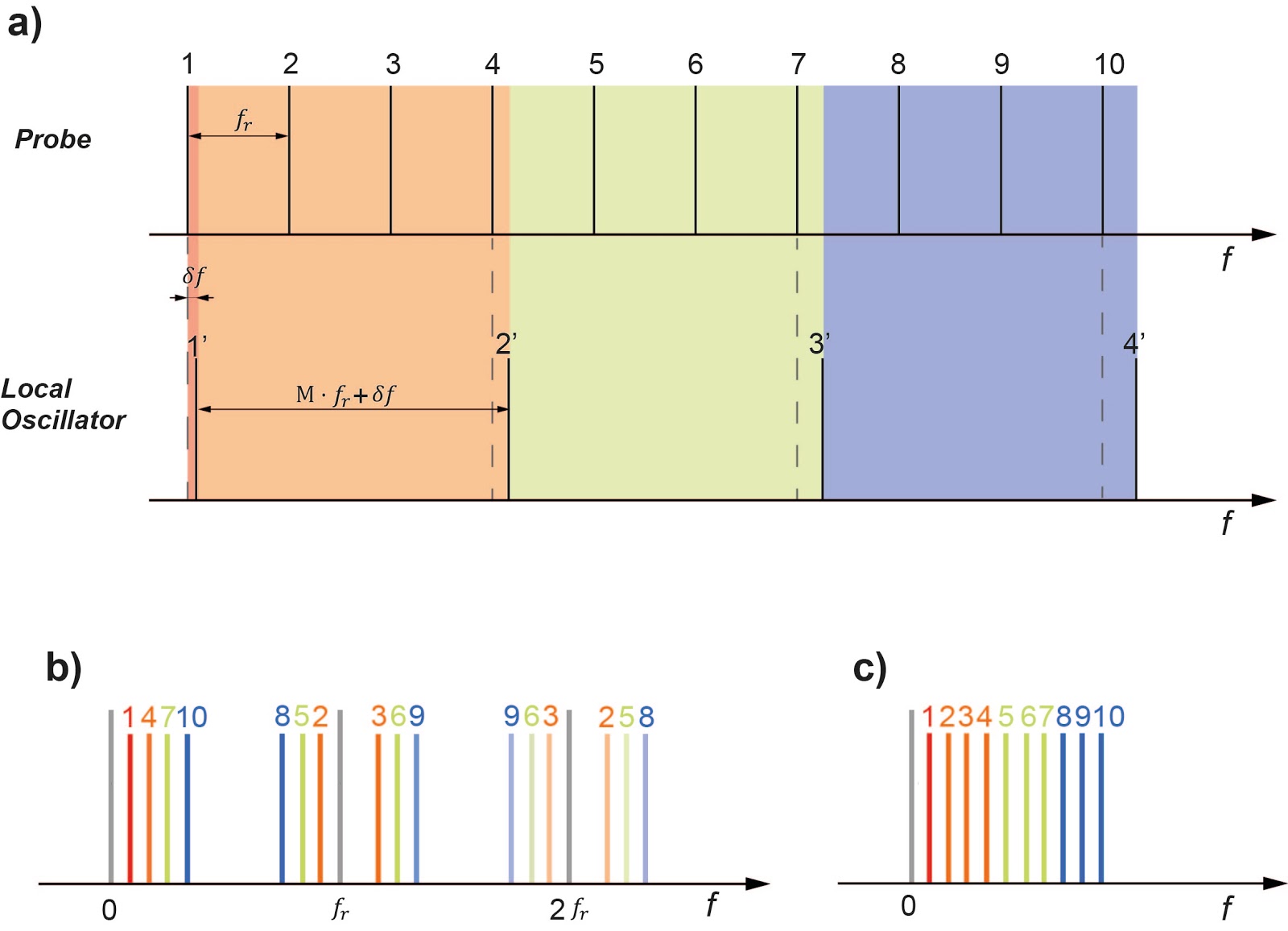


**Figure S1. Frequency domain picture of the dual-comb scheme in a QIR mode. (a) The probe and the LO combs cover similar optical bandwidth but with a different number of teeth. (b) The interference of both combs generates groups of lines (Nyquist zones) on which the response of the probe is encoded. The dim lines around 2**$f_{r}$**show that there is no a unique manner of retrieving that information. (c) After rearranging the lines, a down-converted version of the probe comb can be obtained.**

The optical system to carry out Φ-OTDR based on a dual-comb scheme working in a QIR mode is shown in Figure S2. An ultra-low phase-noise CW laser (CWL), followed by an isolator (Iso), feeds a couple of intensity Mach Zenhder modulators (MZMs). These modulators, driven by an AWG, work as comb generators. The light at the output of each MZMs is boosted by an Erbium-Doped Fiber Amplifier (EDFA). Since the AWG only provides real-valued signals, the spectra of the generated combs show two symmetric complex conjugated sidebands around the laser frequency $f_{0}$ [1]. To avoid aliasing problems in the detection stage, one of the sidebands of each comb is filtered by a single tunable band pass filter (TBPF). For this purpose, two circulators and a bi-directional TBPF are employed, so the probe and LO combs propagate through the filter in opposite directions. In addition, the TBPF also helps to filter out the Amplified Spontaneous Emission (ASE) introduced by the EDFAs. After the comb generation stage, the probe signal is launched into the Fiber Under Test (FUT), where an external perturbation is locally induced. The backscattered signal is collected by a circulator, amplified by an EDFA and properly filtered by a second TBPF to reduce the ASE. The interference of the backscattering signal and LO is detected by a balanced photodetector (BPD). The resulting electrical signal is digitized by a Digital Oscilloscope (Osc.). In order to relax the oscilloscope sampling requirements, an electrical low pass filter is used after the detector.


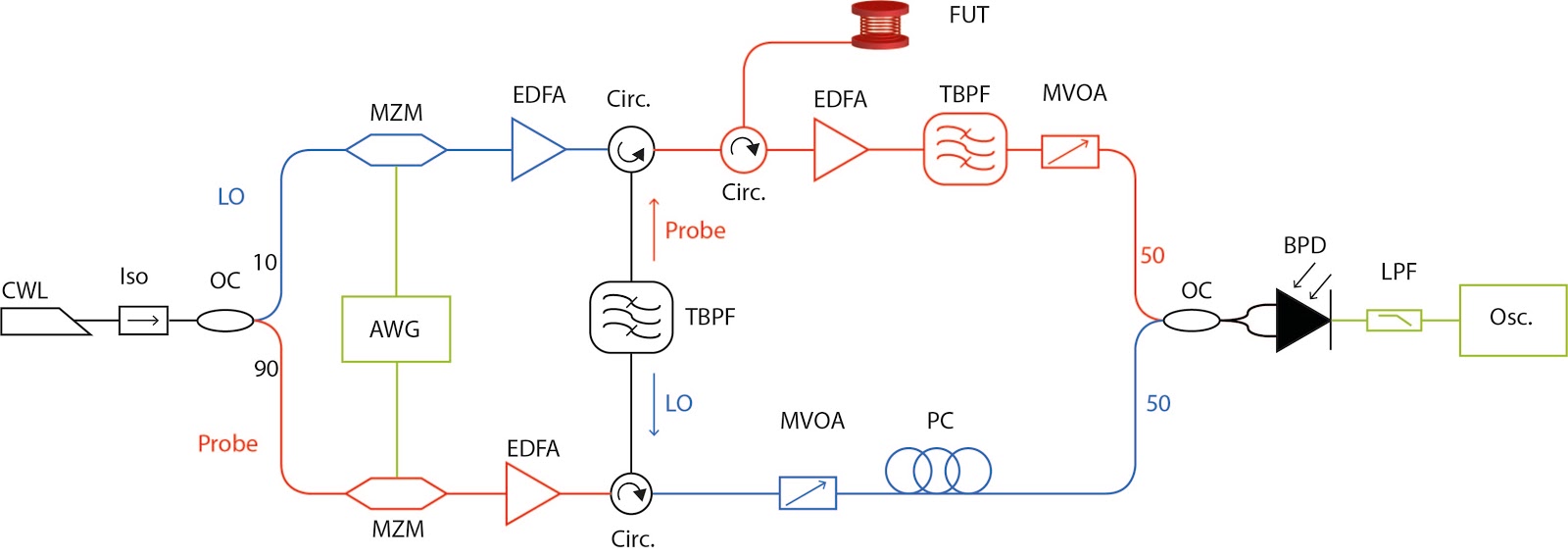


**Figure S2.  Experimental setup to carry out time-expanded Φ-OTDR based on a dual-comb system in a QIR mode. The probe and LO arms are distinguished by the colors red and blue, respectively. The generation and detection stages in the electrical domain are pointed out in green.**

The frequency combs used as probe and LO are generated following the method explained in Section S2. Both combs, after the optical filtering described above, have a bandwidth of 2.5 GHz. The probe comb is made up of 25 000 lines with a line spacing $f_{r}=100$ kHz, so we can interrogate 25 000 individual points over 1 km, thus providing a resolution of 4 cm. Concerning the LO comb, it is composed of 500 lines, that is, the integer factor is $M=50$. The difference $\delta f$ (which fixes the acoustic sampling) is 40 Hz. The low-pass filter used in our experimental setup (see Figure S2) has a cutoff frequency of 6 MHz, which is a small RF bandwidth for a Φ -OTDR sensor with centimeter resolution. In order to avoid the formation of optical pulses and thus increase the SNR, random (but predetermined) spectral phases are allocated to the lines of the probe and LO combs [1]. This can be simply accomplished by making the beat notes involved in the reconstruction algorithm be generated from the interference of pairs of lines with identical (random) phase. As mentioned above, the reconstruction algorithm requires a calibration measurement without FUT [5], which allows to measure the relative phases between the spectral lines of the generated combs. Figure S3 shows a set of traces recorded by the oscilloscope as a function of the position along the fiber, $z$ , which is calculated as $z=ct/2n$ , where $t$ is the round-trip time and $n$ is the refractive index of the fiber. The estimated SNR of the traces is 9.3 dB and they show a good repeatability, as can be observed in the figure inset for a point close to the end of the fiber.


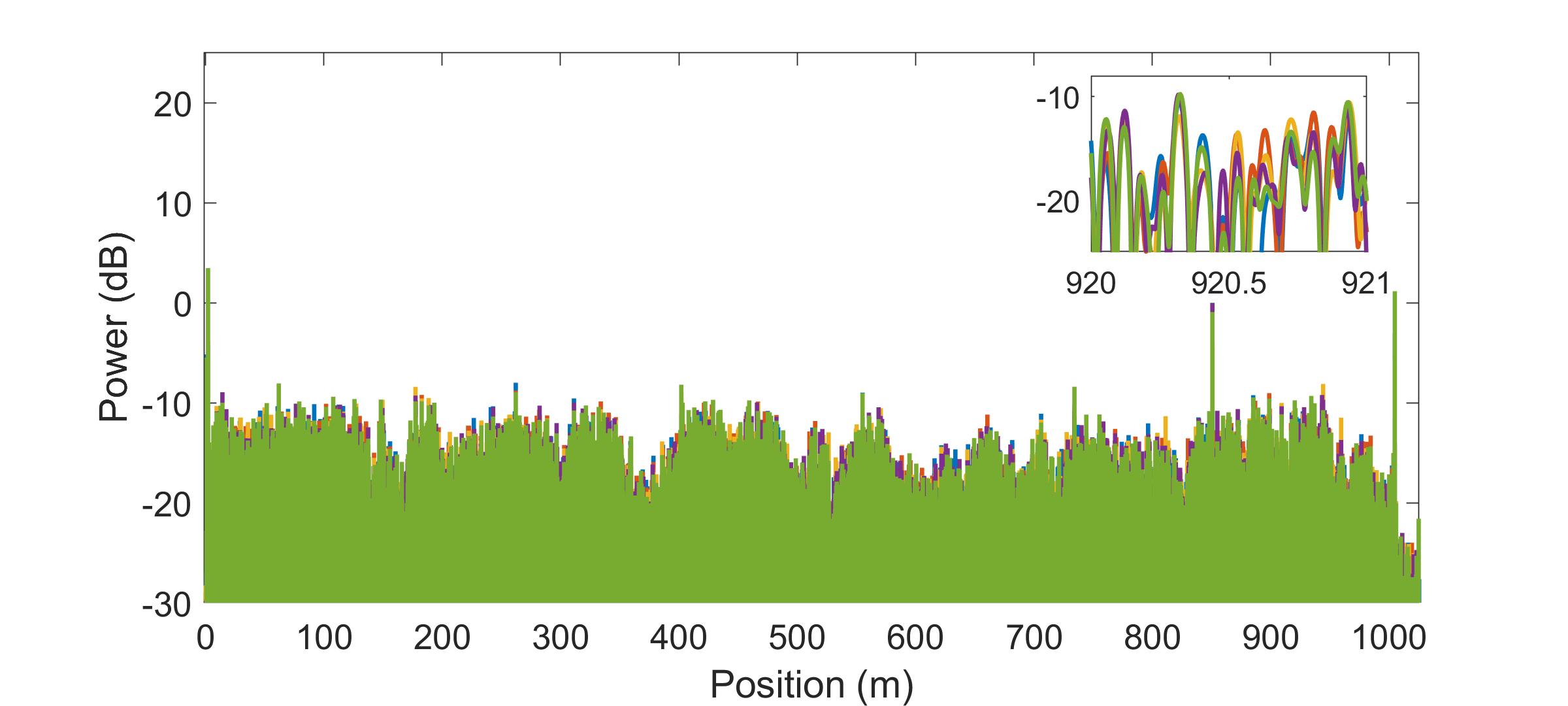


**Figure S3.  Magnitude of the detected traces with an acoustic sampling of 40 Hz when the** **ΦOTDR sensor works in a QIR mode. The inset shows details of several successive traces.**

# *S4. Determination of temperature and strain variations*

The data processing in our time-expanded ΦOTDR sensor leads to the recovery of complex-valued signals, which can be written as a function of the distance. The phase changes in each position of those signals provide the basis for the determination of temperature or strain changes along the fiber. To do so, the phase difference between two fiber points separated by the gauge length $\Delta z$is calculated. This phase difference can be related to the fiber refractive index via:

                                          [S20]

where $k$ is the wavenumber and $n$ is the fiber refractive index.

In the event of a temperature change $\Delta T$, this recorded phase difference will experience a variation , that can be written as:

.                                              [S21]

Note that the terms inside the brackets in this expression are, respectively, the thermo-optic effect coefficient and the coefficient of thermal expansion of the fiber under test. For standard optical fibers (like the one used in this work), these coefficients are known and the temperature can be finally related to using [6]:

,                                        [S22]

where $\Delta z$ and$\lambda_{0}$ are expressed in meters.

Following an analogue reasoning, an induced strain $\Delta\varepsilon$ can be deduced from the phase difference between two points separated by $\Delta z$ through [6]:

,                                           [S23]

where, as before, $\Delta z$ and$\lambda_{0}$ are expressed in meters.

# *S5. Calibration of the time-expanded ΦOTDR with respect to a state of the art chirped-pulse ΦOTDR:*

In order to verify the reliability of the time-expanded ФOTDR, a measurement of the fiber temperature was compared with that of a calibrated chirped-pulse ФOTDR [7], a well- established technology with millikelvin sensitivities and metric resolutions. In order to match the range of operation of the chirped-pulse ФOTDR, the temperature hot-spot was made to have a length of 10 m. The chirped-pulse ФOTDR had a spatial resolution of 6 m, and the time-expanded ФOTDR used a gauge length of 1 m, both sufficiently low in comparison with the size of the applied perturbation, in order to avoid any spatial aliasing problems. The spatio-temporal map of temperature variation measured using the time-expanded ФOTDR is shown in Figure S4a. The temperature profile along the fiber at an instant of maximum temperature change is represented in Figure S4b. Finally, Figure S4c compares the temperature measurement taken by time-expanded ФOTDR (blue) and chirped-pulse ФOTDR (orange). Since the two measurements were not performed at the same time (they correspond to equivalent temperature cycles but separated by a few minutes), the observed signal mismatches (of the order of a few mK) are well within the expectable experimental error.

Additionally, note that in this experiment the current flow is roughly five times smaller than the one used in the Figure 3a of the main body, so the expected temperature change should correspondingly be five times smaller [1].


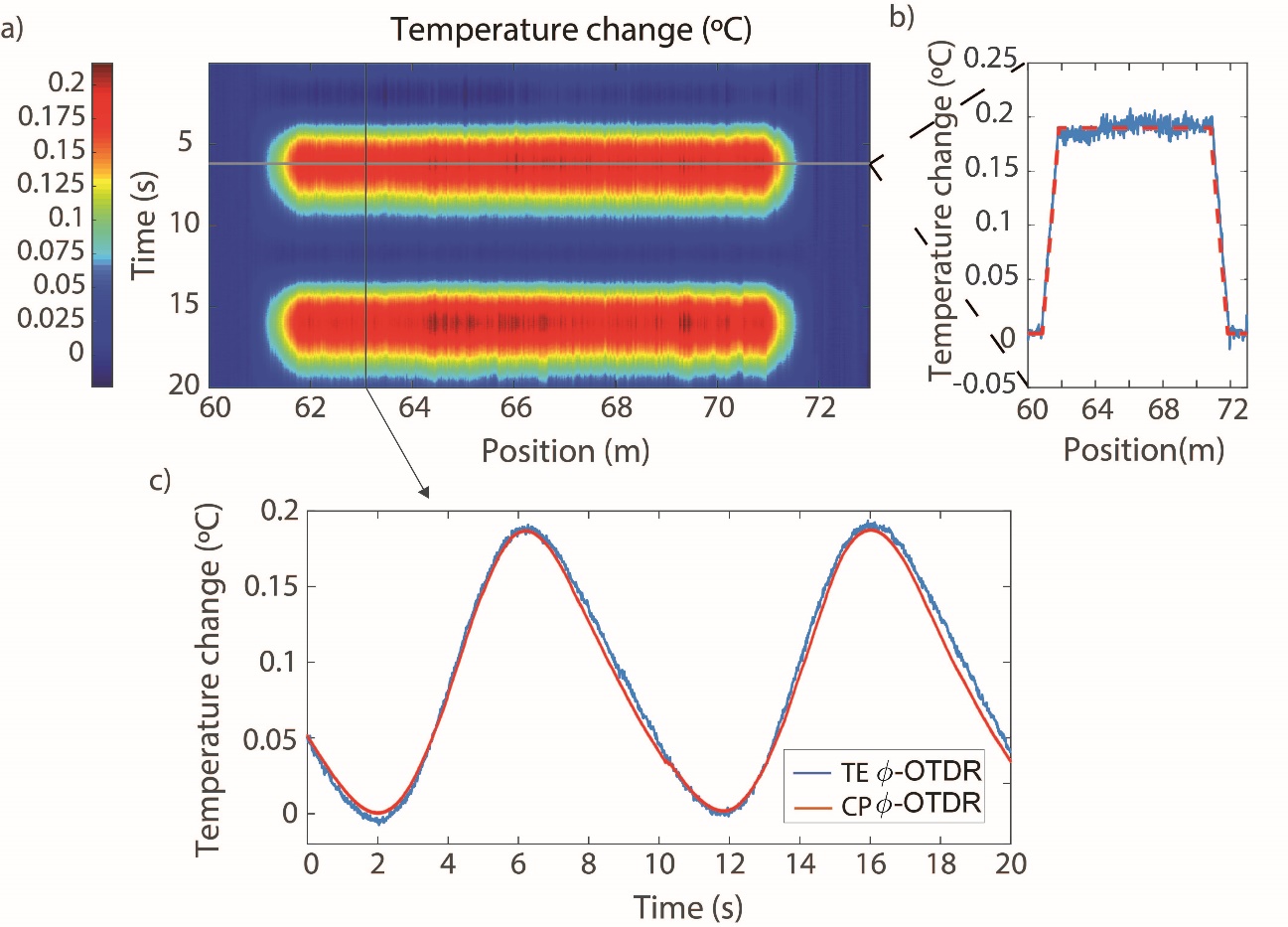


**Figure S4. Comparison of a temperature measurement performed by time-expandedФOTDR with the result obtained using a calibrated chirped-pulse ФOTDR system, demonstrating the reliability/accuracy of the proposed sensor. (a) 10 meter hot-spot measured by time-expanded ФOTDR. (b) the corresponding spatial temperature distribution, matching the expected profile. (c) Comparison of the temperature cycles obtained by time-expanded ФOTDR with those measured by the calibrated chirped-pulse ФOTDR system, showing good agreement.**

# *Referencies*

1. Soriano-Amat, M. *et al*. Common-path dual-comb spectroscopy using a single electro-optic modulator. *Journal of Lightwave Technology* **38,** 5107-5115 (2020).
2. Klee, A. *et al*. Characterization of semiconductor-based optical frequency comb sources using generalized multiheterodyne detection. *IEEE Journal of Selected Topics in Quantum Electronics* **19**, 1100711 (2013).
3. Coddington, I., Newbury, N. & Swann, W. Dual-comb spectroscopy. *Optica* **3**, 414-426 (2016).
4. Klee, A. *et al*. Generalized Spectral Magnitude and Phase Retrieval Algorithm for Self-Referenced Multiheterodyne Detection. *Journal of Lightwave Technology* **31**, 3758–3764 (2013).
5. Hébert, N. B. *et al*. Coherent dual-comb interferometry with quasi-integer-ratio repetition rates. *Optics Express* **22**, 29152-29160 (2014).
6. Koyamada, Y. *et al*. Fiber-optic distributed strain and temperature sensing with very high measurand resolution over long range using coherent OTDR. *Journal of Lightwave Technology* **27**, 1142-1146 (2009).
7. Pastor-Graells, J. *et al*. Single-shot distributed temperature and strain tracking using direct detection phase-sensitive OTDR with chirped pulses. *Optics Express* **24**, 13121-13133 (2016).
